# Intervention effects of physical exercise on negative emotional symptoms in college students: a three-level meta-analysis

**DOI:** 10.3389/fpubh.2026.1911631

**Published:** 2026-10-22

**Authors:** Zhenyao Wang, Lingling Dong, Yan Rao, Haiyan Wen, Cong Liu, Min Huang

**Affiliations:** 1 School of Sport, Loughborough University, Loughborough, United Kingdom; 2 Department of Psychiatry, Shanghai Baoshan District Mental Health Center, Shanghai, China; 3 Shanghai Normal University Tianhua College, Shanghai, China; 4 School of Physical Education & Health, Shanghai University of International Business and Economics, Shanghai, China; 5 School of Physical Education, Shanghai University of Sport, Shanghai, China; 6 School of Physical Education and Health, Shanghai Lixin University of Accounting and Finance, Shanghai, China

**Keywords:** anxiety, depression, meta-analysis, physical exercise, stress

## Abstract

**Objective:**

To systematically evaluate the effects of physical exercise on negative emotional symptoms in college students and examine potential moderators and dose–response relationships.

**Methods:**

Five English-language databases were searched from inception to March 22, 2026. Risk of bias was assessed using the Risk of Bias 2.0 (RoB 2.0) tool, and certainty of evidence using the Grading of Recommendations Assessment, Development and Evaluation (GRADE) approach. Hedges’ g values with 95% confidence intervals (CIs) and prediction intervals (PIs) were pooled using three-level random-effects models. Influence diagnostics, sensitivity analyses, subgroup analyses, publication bias tests, and dose–response analyses were conducted.

**Results:**

Forty-three studies were included. The full-dataset three-level model, retained as the primary analysis, showed a non-significant overall effect (Hedges’ *g* = −1.48, 95% CI −3.21 to 0.26, *p* = 0.094; 95% PI −12.95 to 9.99). Two highly influential effect sizes from one study were identified. Excluding these effects, the influence-adjusted analysis showed a significant effect (Hedges’ *g* = −0.56, 95% CI −0.73 to −0.39, *p* < 0.001; 95% PI −1.45 to 0.33). In influence-adjusted exploratory analyses, baseline population characteristics, geographic region, and outcome measurement instrument were significant moderators, whereas symptom category, exercise modality, frequency, session duration, intervention duration, and publication year were not. No significant linear or nonlinear dose–response relationships were found for weekly or total exercise duration. Overall certainty of evidence was very low.

**Conclusion:**

The full-dataset analysis did not provide statistically significant evidence of an overall effect. Although the influence-adjusted analysis suggested a potential benefit, the result was sensitive to influential observations and certainty of evidence was very low. Accordingly, no definitive conclusion regarding efficacy can be drawn. Potential moderator findings require confirmation in future high-quality randomized controlled trials.

## Background

The university years represent a critical developmental period during which individuals transition from late adolescence to early adulthood, and they also constitute a stage in which mental health problems are particularly likely to emerge. Owing to pressures related to role transition, academic demands, employment concerns, financial burden, and interpersonal adjustment, college students are especially vulnerable to negative emotional symptoms such as depression, anxiety, and stress (1). These symptoms not only undermine learning engagement, academic performance, and social adjustment, but may also further impair sleep, quality of life, and overall physical and mental well-being, and may even increase the risk of self-harm, suicide, and progression to more severe psychiatric disorders (2, 3).

Current evidence indicates that the mental health burden among college students remains high worldwide. Recent meta-analytic evidence suggests that approximately one-third to two-fifths of college students experience depressive, anxiety, or stress symptoms, although prevalence estimates vary across populations and assessment methods (4, 5). Moreover, these symptoms frequently co-occur, further increasing their impact on students’ health, academic adaptation, and social functioning (6).

Given the high prevalence of negative emotional symptoms in college students, it is of clear practical importance to identify interventions that are safe, accessible, low-cost, and feasible for implementation on campus. Compared with pharmacological treatment and formal psychotherapy, exercise has several advantages, including fewer side effects, easier implementation, and better integration into daily life and university settings; accordingly, it has gradually become an important non-pharmacological approach for promoting mental health in college students (7). Existing research suggests that exercise may exert beneficial effects on negative emotional symptoms through multiple pathways, including modulation of neurotransmitter function, regulation of the hypothalamic–pituitary–adrenal axis, enhancement of positive affective experiences, and improvement of self-efficacy (8, 9). Nevertheless, the current evidence base still has several limitations. First, outcome measures vary substantially across studies, with some focusing on depression, others on anxiety or stress, making it difficult to obtain an integrated estimate of the overall effects of exercise on negative emotional symptoms in college students (10, 11). Second, primary studies often report multiple scales, dimensions, or effect sizes simultaneously. Under such circumstances, traditional meta-analysis is constrained by the assumption of effect-size independence, which may lead to information loss and reduce the accuracy of pooled estimates (12).

In addition, conclusions remain inconsistent regarding the optimal exercise modality and appropriate exercise dose for improving negative emotional symptoms in college students (7, 10). Different forms of exercise, such as aerobic exercise, resistance training, mind–body exercise, and multicomponent exercise, may differ in their mechanisms, exercise load, and emotion-regulating effects. Likewise, exercise frequency, session duration, intervention duration, weekly exercise duration, and total exercise duration may all influence intervention outcomes (13). Without systematic synthesis, it is difficult not only to determine the true intervention effects of exercise on negative emotional symptoms in college students, but also to provide a sound basis for developing evidence-informed exercise programs in campus mental health practice.

Depression, anxiety, and stress were integrated under the broader framework of negative emotional symptoms because, although they represent distinct psychological constructs, they share a substantial common component of negative affect and general psychological distress and are frequently conceptualized as closely related internalizing symptom domains. These symptoms also commonly co-occur in college students and may partly reflect overlapping emotional and stress-response processes. In addition, several widely used instruments, such as the Depression Anxiety Stress Scales, assess depression, anxiety, and stress within a unified measurement framework. Therefore, the term “negative emotional symptoms” was used in the present study as an overarching analytical construct rather than to imply that depression, anxiety, and stress are conceptually identical. To preserve construct-specific information, subgroup analyses by symptom category were conducted to examine whether intervention effects differed across depression, anxiety, and stress.

Against this background, the present study employed a three-level meta-analytic approach to systematically synthesize evidence on the effects of physical exercise on depressive, anxiety, and stress symptoms in college students. By accounting for the dependency among multiple effect sizes reported within the same study, this approach allows a more appropriate estimation of the overall effect of physical exercise. In addition, this study examined the moderating roles of population characteristics, symptom category, exercise modality, and exercise dose, and explored the dose–response relationships of weekly exercise duration and total exercise duration. The ultimate aim was to provide more robust evidence to inform precise and scalable exercise-based interventions for negative emotional symptoms in college students.

## Methods

This meta-analysis was conducted in strict accordance with the methodological recommendations of the Cochrane Handbook for Systematic Reviews of Interventions (14). The study protocol was prospectively registered in the International Prospective Register of Systematic Reviews (PROSPERO; registration number: CRD420261334375), in order to enhance transparency and reduce the risk of selective reporting bias. The review was planned and reported with reference to the Preferred Reporting Items for Systematic Reviews and Meta-Analyses (PRISMA) statement (15), so as to ensure methodological rigor, completeness, and reproducibility.

### Eligibility criteria

Participants: The target population was college students, including junior college students, undergraduates, and postgraduates. Eligible samples could consist either of college students with depressive, anxiety, or stress symptoms, or of general college student populations without clearly defined screening thresholds. The original study was required to report at least one outcome related to depression, anxiety, or stress and to provide extractable data.

Intervention: The intervention group had to receive an exercise intervention, defined as a planned, organized, or structured program of physical activity, such as aerobic exercise, resistance training, mind–body exercise, multicomponent exercise, or other forms of exercise training. No strict restrictions were imposed on exercise modality, intensity, frequency, session duration, or intervention duration, provided that exercise was clearly the core component of the intervention.

Comparison: The control group was required to receive a non-exercise condition, including blank control, maintenance of usual daily life, wait-list control, health education, usual care, or other non-exercise interventions. Studies were excluded if the control group also received a systematic exercise intervention, as this would preclude a valid comparison.

Outcomes: The outcomes of interest were negative emotional symptoms, including depression, anxiety, and stress. In the primary analysis, depression-, anxiety-, and stress-related outcomes were first combined into an overall effect for negative emotional symptoms. Subgroup analyses were then conducted separately for depression, anxiety, and stress. This analytical strategy was used to estimate the overall effect of physical exercise on negative emotional symptom burden while retaining the ability to examine whether intervention effects differed across specific symptom domains. The symptom category was therefore included as a moderator in subgroup analyses. Eligible studies had to report at least one of these outcomes and provide sufficient data for effect size calculation. Depression-related measures included, for example, the Beck Depression Inventory (BDI), the Center for Epidemiologic Studies Depression Scale (CES-D), the Self-Rating Depression Scale (SDS), and the Patient Health Questionnaire-9 (PHQ-9); anxiety-related measures included the Self-Rating Anxiety Scale (SAS) and the State–Trait Anxiety Inventory (STAI); stress-related measures included the Perceived Stress Scale (PSS). Other multidomain instruments included the Depression Anxiety Stress Scales (DASS-21/DASS-42), the Hospital Anxiety and Depression Scale (HADS), and the Symptom Checklist-90 (SCL-90). When a study reported more than one eligible outcome, each outcome was extracted as a separate effect size, and the statistical dependence among these effect sizes was handled in the three-level meta-analysis.

Study design: Only randomized controlled trials (RCTs) were included. Studies were required to report complete between-group comparative data, or at least provide the raw statistics needed to calculate effect sizes, such as means, standard deviations, and sample sizes.

### Exclusion criteria

Studies were excluded if: (1) the participants were not college students; (2) exercise was not the core component of the intervention, or exercise was only a minor part of a multicomponent intervention and its independent effect could not be identified; (3) the control condition also involved a clearly defined exercise intervention; (4) the study did not report depression-, anxiety-, or stress-related outcomes, or reported only other outcomes such as sleep, fatigue, or quality of life without providing data on negative emotional symptoms; (5) insufficient data were reported for effect size calculation and the necessary data could not be obtained after contacting the authors; (6) the study was not an RCT, such as an observational study, single-group pre-post study, review, conference abstract, or case report; (7) the study was a duplicate publication, in which case only the most complete report was retained; or (8) the article was published in a language other than English.

### Literature search and study selection

A systematic search was conducted in five English-language electronic databases—Web of Science, PubMed, Embase, the Cochrane Library, and Scopus—for RCTs examining exercise interventions for negative emotional symptoms in college students. The search covered each database from inception to March 22, 2026. Both controlled vocabulary terms and free-text terms were used, combined with the Boolean operators AND and OR. The main search terms included “physical activity,” “exercise,” “physical fitness,” “yoga,” “tai chi,” “university students,” “college students,” “mental health,” “psychological health,” “anxiety,” “depression,” and “stress.” To minimize the risk of missing relevant studies, the reference lists of included articles and relevant reviews were also hand-searched. The full search strategy is presented in Supplementary Table 1.

Study selection was performed independently by two reviewers. First, all retrieved records were imported into EndNote X9 and duplicates were removed. Titles and abstracts were then screened to exclude clearly irrelevant studies. Full texts of potentially eligible articles were subsequently reviewed to determine final inclusion. Any disagreement between the two reviewers was resolved through discussion with a third reviewer until consensus was reached.

### Data extraction and coding

Two reviewers independently extracted information on study characteristics, intervention characteristics, and outcome data from the included studies. Extracted study characteristics included first author, publication year, country, sample size, mean age, proportion of female participants, population characteristics, and outcome measures. Intervention characteristics included exercise modality, session duration, exercise frequency, intervention duration, exercise intensity, and the content of the control condition. The extracted quantitative data were post–pre change scores, expressed as means and standard deviations. Effect sizes were calculated as Hedges’ g based on between-group differences in pre–post change scores. For each group, the mean change was calculated as the post-intervention mean minus the baseline mean. When the standard deviation of the change score was not reported, it was estimated using the following formula: $SD_{\text{change}}=\sqrt{SD_{\text{baseline}}^{2}+SD_{\text{post}}^{2}–2\times r\times SD_{\text{baseline}}\times SD_{\text{post}}}$ . A pre–post correlation coefficient of r = 0.50 was assumed in the primary analysis. To assess the robustness of the effect-size estimates to this assumption, sensitivity analyses were additionally conducted using alternative pre–post correlation coefficients of *r* = 0.30 and *r* = 0.70. Disagreements between the two reviewers were resolved through consultation with a third reviewer.

To explore potential sources of heterogeneity, moderator analyses were further conducted. Based on baseline participant status in the original studies, samples were coded into three groups: a negative emotional symptom group, a general college student group, and an other special population group. Studies that explicitly recruited college students with depression, anxiety, or stress problems, or participants who met predefined thresholds on relevant screening tools, were coded as the negative emotional symptom group. Studies that recruited ordinary college students without requiring participants to meet any screening threshold were coded as the general college student group. Studies focusing on college students with other specific health risks or behavioral problems, such as fatigue, mobile phone addiction, or obesity, were coded as the other special population group.

Depression-, anxiety-, and stress-related outcomes were collectively coded as negative emotional symptoms, and further classified into three dimensions: depression, anxiety, and stress. Exercise modality was coded as aerobic exercise, resistance training, mind–body exercise, multicomponent exercise, and other exercise modalities. Session duration was coded into four categories: 10–30 min, 31–60 min, >60 min, and Unclear. Intervention duration was coded as 1–7 weeks or 8–16 weeks. Exercise intensity was not coded because a number of studies did not report it clearly.

In addition, based on the exercise prescription information reported in the original studies, two continuous variables—weekly exercise duration and total exercise duration—were constructed for moderator and dose–response analyses. Weekly exercise duration was calculated as session duration × exercise frequency per week, and total exercise duration as weekly exercise duration × intervention duration. For studies that reported only a range for session duration rather than an exact value (e.g., 30–60 min), the midpoint of the interval was used as an approximate estimate. All coding procedures were conducted independently by two reviewers, and disagreements were resolved through discussion with a third reviewer until consensus was achieved. To further explore study-level heterogeneity, geographic region, outcome measurement instrument, and publication year were examined as additional exploratory moderators. Geographic region was categorized as Asia, Europe, North America, or Other. Outcome measures were grouped into the BDI family, DASS family, PSS family, STAI family, Zung self-rating scales, or other instruments. Publication year was analyzed as a continuous variable.

### Risk of bias of included studies

The methodological quality of the included studies was assessed using the Risk of Bias 2.0 (RoB 2.0) tool. This instrument evaluates each study across five domains: bias arising from the randomization process, bias due to deviations from intended interventions, bias due to missing outcome data, bias in the measurement of the outcome, and bias in the selection of the reported result. Based on judgments across these domains, an overall risk-of-bias rating was assigned to each study (16).

Risk-of-bias assessment was conducted independently by two reviewers. On the basis of the methodological information reported in the original articles, each domain was rated as “low risk,” “some concerns,” or “high risk.” Any disagreements between the two reviewers were resolved through discussion; when consensus could not be reached, a third reviewer was consulted for adjudication.

### Statistical analysis

Because some primary studies reported multiple negative emotional symptoms, the effect sizes were likely to be statistically dependent within studies. To account for this within-study dependency, a three-level random-effects meta-analysis was conducted, in which variance was partitioned into sampling variance (Level 1), within-study variance (Level 2), and between-study variance (Level 3) (12, 17). Effect sizes were expressed as Hedges’ g, together with their 95% confidence intervals (95% CI) and 95% prediction intervals (95% PI); negative values indicated improvement in negative emotional symptoms. Heterogeneity was evaluated using Cochran’s Q test, variance decomposition, and likelihood ratio tests (18). Moderator analyses were further performed using three-level meta-regression. In addition to the prespecified population and intervention characteristics, geographic region and outcome measurement instrument were examined as exploratory categorical moderators, while publication year was examined using linear and natural spline nonlinear meta-regression. Both linear and natural spline nonlinear models were also fitted for weekly exercise duration and total exercise duration to examine potential dose–response relationships (17, 19). Robustness analyses included cluster-robust variance estimation using the CR2 adjustment, leave-one-study-out (LOSO) analysis, leave-one-effect-out (LOO) analysis, and influence diagnostics (20, 21). Influence diagnostics were conducted to identify statistically extreme and highly influential effect sizes before the final inferential analyses. An effect size was considered highly influential when it showed an extreme standardized residual and/or was identified by multiple influence indices, including Cook’s distance, hat values, and leave-one-out diagnostics. Effect sizes were not excluded solely because of their magnitude or because of their impact on statistical significance. When an effect size was simultaneously identified as statistically extreme and exerted a disproportionate influence on the pooled estimate, confidence interval, prediction interval, and heterogeneity structure, an additional influence-adjusted analysis was performed after excluding these observations. The model based on the full dataset was retained as the primary analysis of the overall pooled effect. Given the substantial influence of these observations on model stability and heterogeneity, subsequent subgroup, moderator, and dose–response analyses were primarily interpreted using the influence-adjusted dataset. Corresponding analyses based on the full dataset were additionally conducted to assess the sensitivity of these exploratory findings and are reported in Supplementary material. Publication bias was assessed using funnel plots, Egger’s test, trim-and-fill, and a multilevel Egger test (22, 23). The certainty of evidence was evaluated using the Grading of Recommendations Assessment, Development and Evaluation (GRADE) approach with GRADEpro (24–27). Further details are provided in Supplementary material.

## Results

### Study selection

A total of 13,570 records were identified through the database search. After removal of 3,866 duplicates, 9,704 records remained for title and abstract screening. Following the initial screening, 9,589 clearly irrelevant records were excluded, leaving 115 articles for full-text review. The full texts of these 115 articles were retrieved and assessed for eligibility. Of these, 12 studies were excluded because the outcome measures did not meet the inclusion criteria, 16 were reviews or conference abstracts, 9 did not provide accessible data, 6 were not randomized controlled trials, 14 were duplicate publications, 7 were non-English articles, and 8 did not involve college student populations. Ultimately, 43 studies were included in the meta-analysis (28–70). The study selection process is presented in Figure 1.

**Figure 1:**
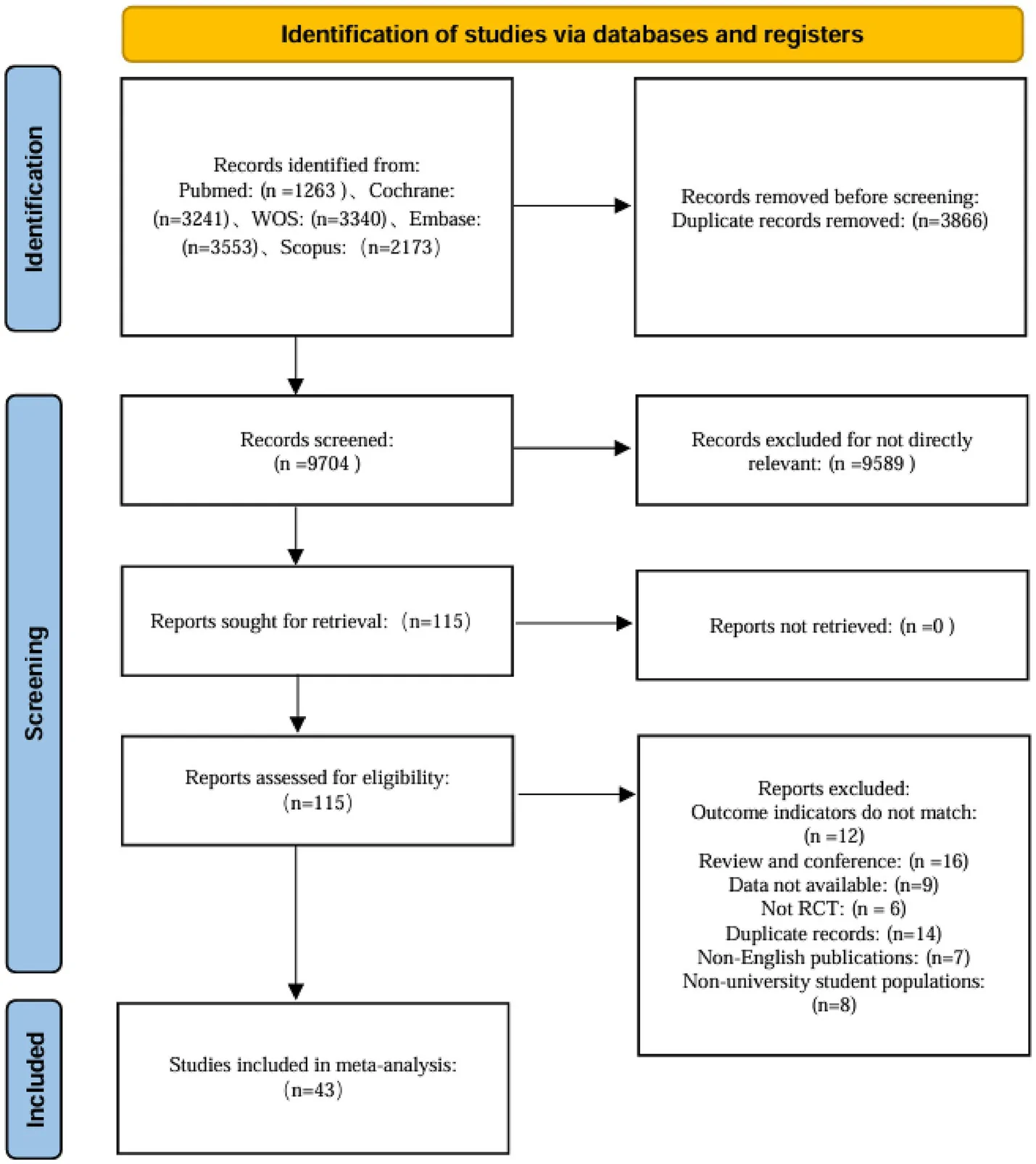
Flow diagram of study selection.

### Characteristics of included studies

A total of 43 studies were included, comprising 3,199 participants. The included studies were conducted across 16 countries, with the largest number from China (14 studies), followed by Türkiye (6 studies). Sample sizes ranged from 17 to 206 participants per study. The mean age of participants was generally concentrated within the typical college-age range; among studies that reported mean age values, the average age ranged from 18.41 to 26.06 years, although a small number of studies did not report mean age. Female participants accounted for a relatively large proportion of the total sample. In terms of population characteristics, the included studies involved not only general college student samples, but also several specific subgroups with different baseline characteristics, including students with depressive symptoms, students with anxiety symptoms, students with mental health problems or psychological disorders, students with fatigue, students with mobile phone addiction, and students with overweight or obesity. These features suggest a certain degree of heterogeneity in the included samples. Detailed characteristics of the included studies are presented in Table 1.

**Table 1:** Basic characteristics of the included studies.

Included study	Country	Sample size	Mean age	Female proportion	Population characteristics
Abavisani et al. (28)	Iran	62	23.77	Unclear	General college students
Akandere and Demir (29)	Turkey	120	20–24	50%	General college students
Alsaraireh and Aloush (30)	Jordan	181	22	62%	General college students
Aşçı (31)	Turkey	40	21.28	100%	General college students
Barğı (33)	Turkey	31	19.97	90.32%	General college students
de Vries et al. (38)	Netherlands	99	20.8	80.80%	College students with fatigue
Dinani et al. (39)	Iran	64	21.50	Unclear	General college students
Eather et al. (40)	Australia	53	20.38	63.60%	General college students
Ghorbani et al. (43)	Iran	30	26.06	100%	General college students
Hemat-Far et al. (44)	Iran	20	18–25	100%	College students with depressive symptoms
Kim et al. (47)	South Korea	54	19–25	100%	General college students
Kim et al. (46)	USA	18	24.83	Unclear	College students with anxiety symptoms
Li et al. (48)	China	206	20.78	82.5%	General college students
Li et al. (49)	China	27	22.55	100%	College students with anxiety symptoms
López-Rodríguez et al. (51)	Spain	95	22.07	74.7%	General college students
Paolucci et al. (52)	Canada	55	21	71.0%	General college students
Papp et al. (53)	Sweden	44	25	86.36%	General college students
Philippot et al. (54)	Belgium	28	20.81	89.29%	College students with psychological disorders
Roth and Holmes (55)	USA	55	18.9	50.91%	College students with psychological disorders
Sadeghi et al. (56)	Iran	46	20.99	21.74%	College students with depressive symptoms
Saltan and Ankaralı (57)	Turkey	92	19.03	81.85%	General college students
Wan Yunus et al. (59)	Malaysia	36	22.89	86.11%	General college students
Xiao et al. (62)	China	100	19.29	71.0%	College students with psychological disorders
Yiğiter and Hardee (63)	Turkey	60	19.07	100.0%	General college students
Zhang et al. (66)	China	18	23.35	72.22%	College students with psychological disorders
Zhang and Jiang (68)	China	78	19.2	100.0%	General college students
Zhao et al. (69)	China	86	21.2	70.9%	College students with depressive symptoms
Ji et al. (70)	China	198	20.6	67.2%	General college students
(45)	China	197	21.3	41.62%	General college students
Gallego et al. (42)	Spain	125	20.07	57.6%	General college students
Zhang et al. (67)	China	90	20.11	60.0%	College students with mobile phone addiction
Brandão et al. (34)	Portugal	106	21.04	90.6%	General college students
Calazans de Lira et al. (35)	Brazil	17	19.3	64.71%	General college students
Falsafi (41)	USA	67	22.1	86.4%	College students with psychological disorders
Castellote-Caballero et al. (36)	Spain	129	20.29	51.2%	General college students
Suwannakul et al. (58)	Thailand	44	20.38	100%	College students with overweight/obesity
Lin Latt et al. (50)	USA	28	21	100%	General college students
Zhang et al. (65)	China	62	18.41	66.13%	College students with depressive symptoms
Cui et al. (37)	China	56	20.3	64.29%	College students with overweight/obesity
Wang et al. (61)	China	93	19.28	47.31%	General college students
Wang et al. (60)	China	45	20.40	77.78%	General college students
Yun et al. (64)	China	41	19.73	63.05%	College students with depressive symptoms
Aslan et al. (32)	Turkey	103	19.6	58.25%	General college students

The exercise interventions were diverse and included Pilates, dance training, aerobic exercise, Tai Chi, Baduanjin, yoga, resistance training, high-intensity interval training (HIIT), and some ball games and exergaming interventions. Session duration was most commonly 30–90 min, exercise frequency was mainly two to three sessions or three sessions per week, and intervention duration was generally concentrated within 4–16 weeks. Exercise intensity was reported inconsistently across studies. Some studies defined exercise intensity using maximal heart rate (HRmax), heart rate reserve (HRR), repetition maximum (RM), one-repetition maximum (1RM), or rating of perceived exertion (RPE), whereas others did not clearly specify intensity. Control conditions were most commonly usual life or wait-list control, although a small number of studies used active control conditions. Outcome measures mainly included the BDI, STAI, PSS, DASS-21/DASS-42, SAS, SDS, CES-D, HADS, SCL-90, and PHQ-9. Further details are provided in Supplementary Table 2.

### Risk of bias assessment

Of the 43 included studies, 38 were judged to be at high risk of bias, while 5 were rated as some concerns; no study was classified as having an overall low risk of bias. Across domains, bias due to deviations from intended interventions was commonly rated as some concerns, suggesting that in exercise intervention studies, such bias is difficult to avoid because blinding of participants is usually not feasible. Bias in the measurement of outcomes was the main source of methodological concern. This judgment was not based merely on the use of self-report scales, but on the combination of subjective outcome assessment, lack of participant blinding, and the possibility that awareness of group allocation could influence participants’ responses. Detailed domain-level judgments are provided in Supplementary Figure 1. By contrast, the overall situation was relatively better for the randomization process and selection of the reported result, with most studies rated as either low risk or some concerns and comparatively few rated as high risk. Bias due to missing outcome data varied across studies: some studies handled missing data well, whereas others were judged to be at high risk because of loss to follow-up, attrition, or per-protocol analysis. Overall, the methodological quality of the included studies was relatively limited, with the main concerns centering on intervention implementation and bias related to the measurement of subjective outcomes (see Supplementary Figure 1).

### Primary meta-analysis based on the full dataset

A total of 43 studies comprising 118 effect sizes were included in the meta-analysis. The three-level random-effects model showed a non-significant overall effect (Hedges’ *g* = −1.48, 95% CI −3.21 to 0.26, *p* = 0.094; 95% PI −12.95 to 9.99). In contrast, the two-level random-effects model yielded a significant overall effect (Hedges’ *g* = −1.17, 95% CI −1.97 to −0.37, *p* = 0.005; 95% PI −9.84 to 7.50). These results suggested that the pooled estimate was sensitive to model specification and potentially influenced by extreme or highly influential effect sizes.

### Influence analysis and sensitivity analysis

To evaluate the robustness of the findings, CR2-based robust variance estimation as well as LOSO and LOO sensitivity analyses were conducted. The results showed that, in the baseline model, the restricted maximum likelihood estimate was −1.479 (95% CI: −3.213 to 0.256), whereas the CR2-adjusted estimate was −1.479 (95% CI: −3.245 to 0.288). The high degree of consistency between these estimates suggests that the primary model was not sensitive to robust variance correction. Further LOSO analysis showed that the CR2-adjusted pooled effect ranged from −1.527 to −0.561, with a maximum absolute change of 0.918, indicating that the largest change in the overall effect occurred after removal of one study (47). This suggests that the overall findings may have been substantially influenced by an individual study at the study level. In contrast, the LOO analysis showed a maximum absolute change of 0.118, indicating that the largest change in the pooled effect after removing a single effect size was relatively limited (47). Sensitivity analyses were also conducted to examine the influence of the assumed pre–post correlation coefficient used to estimate change-score standard deviations. Using the full dataset of 118 effect sizes, the three-level pooled effect remained statistically non-significant when *r* = 0.30 (Hedges’ *g* = −1.08, 95% CI −2.20 to 0.04, *p* = 0.059; 95% PI −8.47 to 6.31) and when *r* = 0.70 (Hedges’ *g* = −1.59, 95% CI −3.24 to 0.06, *p* = 0.059; 95% PI −12.51 to 9.33). These findings were consistent with the primary analysis using *r* = 0.50 (Hedges’ *g* = −1.48, 95% CI −3.21 to 0.26, *p* = 0.094; 95% PI −12.95 to 9.99), indicating that the statistical conclusion of the full-dataset three-level model was robust to plausible alternative assumptions regarding the pre–post correlation coefficient.

In addition, influence diagnostics were conducted to further examine the impact of potential outliers on the robustness of the findings. Two effect sizes were identified as statistically extreme and highly influential observations, and both originated from the same study (47) (see Figure 2). These two effect sizes were not merely large in magnitude; rather, they exerted a disproportionate influence on the pooled estimate, confidence interval, prediction interval, and heterogeneity structure. After excluding these two highly influential effect sizes, the influence-adjusted three-level random-effects model showed a significant overall effect (Hedges’ *g* = −0.56, 95% CI: −0.73 to −0.39, *p* < 0.001; 95% PI: −1.45 to 0.33). Similarly, the influence-adjusted two-level random-effects model also indicated a significant overall effect (Hedges’ *g* = −0.59, 95% CI: −0.71 to −0.47, *p* < 0.001; 95% PI: −1.40 to 0.22) (see Figure 3). Because these two effect sizes had a disproportionate impact on the stability and interpretability of the pooled estimate and substantially affected the heterogeneity structure, the influence-adjusted dataset was used for the primary interpretation of subsequent exploratory subgroup, moderator, and dose–response analyses. The full-dataset model remained the primary analysis of the overall pooled effect. Corresponding full-dataset moderator analyses were additionally conducted to evaluate the sensitivity of these exploratory findings and are reported in Supplementary material.

**Figure 2:**
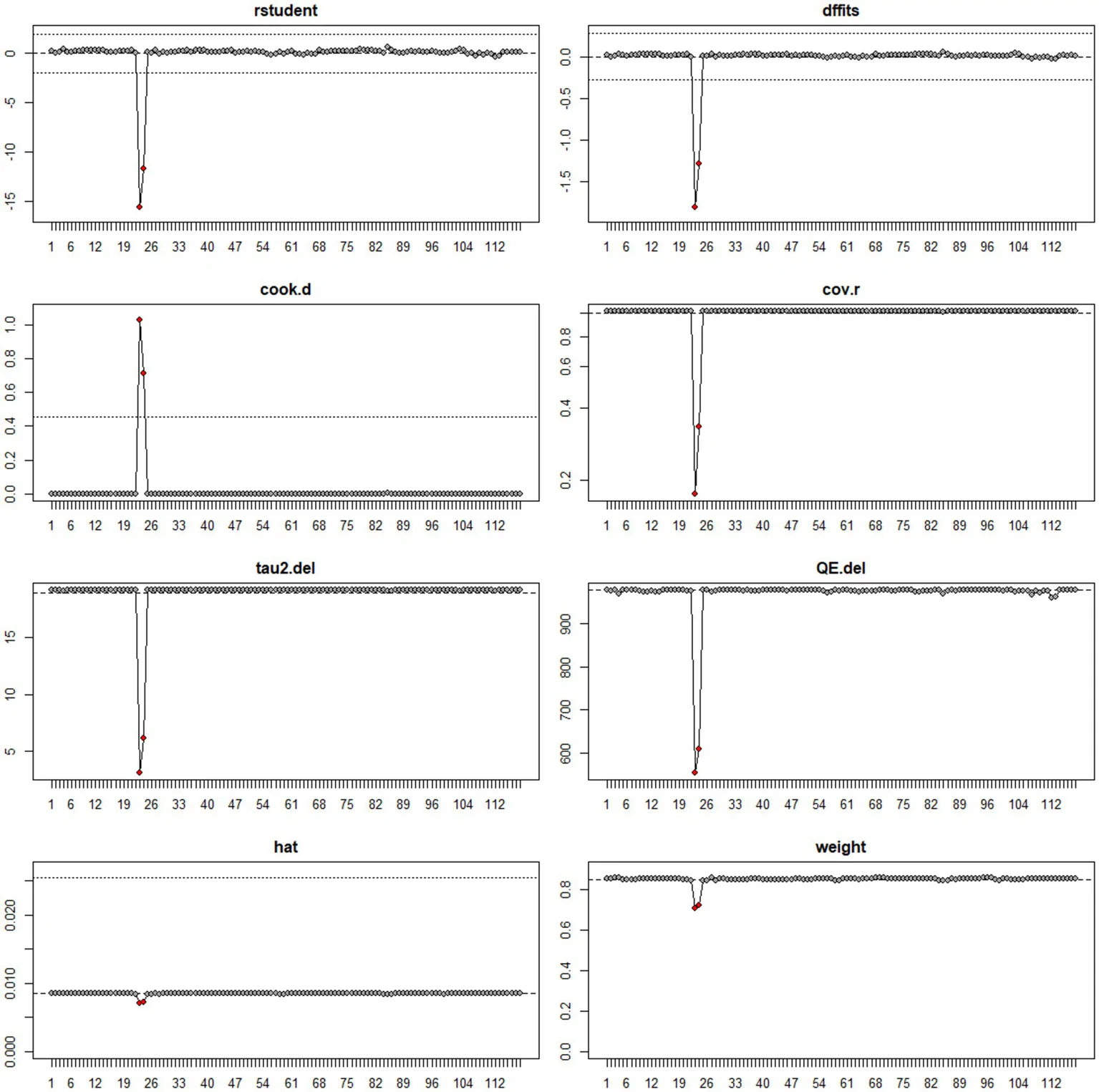
Influence analysis. Each point represents one effect size in the influence diagnostics. Rstudent, studentized residual; dffits, change in the fitted value after deletion of an individual effect size; cook.d, Cook’s distance; cov.r, covariance ratio; tau2.del, estimated heterogeneity variance after deletion of an individual effect size; QE.del, residual heterogeneity statistic after deletion; hat, leverage (hat value); and weight, relative model weight. Larger absolute studentized residuals, DFFITS, Cook’s distances, and leverage values, as well as substantial changes in the covariance ratio, heterogeneity estimates, or residual heterogeneity statistic after deletion, indicate greater potential influence. The two highly influential effect sizes from Kim et al. (47) are highlighted.

**Figure 3:**
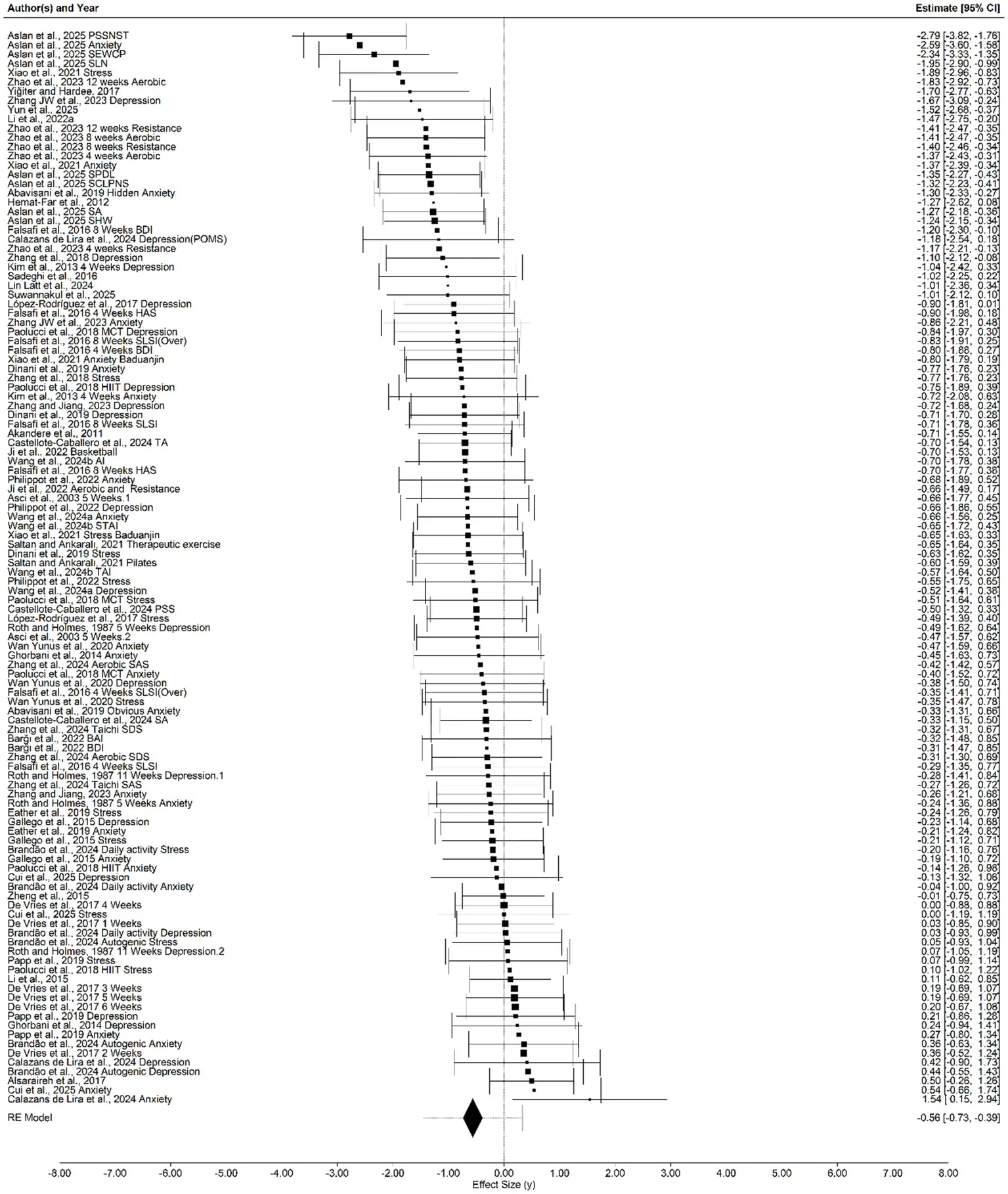
Forest plot of the effects of physical exercise on negative emotional symptoms in college students. Squares represent individual effect sizes, with square size proportional to the inverse-variance weight, and horizontal lines indicate 95% confidence intervals (CIs). The diamond represents the pooled Hedges’ g estimate and its 95% CI from the influence-adjusted three-level random-effects model. Negative Hedges’ g values indicate greater reductions in negative emotional symptoms in the exercise group than in the control group. Text following the author and publication year identifies the relevant outcome, intervention condition, or assessment time point. BDI, Beck Depression Inventory; BAI, Beck Anxiety Inventory; HIIT, high-intensity interval training; MCT, moderate-intensity continuous training; POMS, Profile of Mood States; SDS, Self-Rating Depression Scale; SAS, Self-Rating Anxiety Scale; PSS, Perceived Stress Scale; STAI, State–Trait Anxiety Inventory; SA, state anxiety; TA, trait anxiety. Additional study-specific abbreviations are reported as in the original studies and are detailed in Supplementary Table 2.

### Heterogeneity analysis

In the influence-adjusted dataset, Cochran’s Q test indicated significant overall heterogeneity [Q(115) = 183.88, *p* < 0.001], suggesting substantial variability across effect sizes. To further identify the sources of heterogeneity, a three-level random-effects meta-analysis model was fitted to decompose the total variance into different levels. The results showed that sampling variance accounted for 58.21% of the total variance, within-study variance for 2.18%, and between-study variance for 41.79%. The likelihood ratio test (LRT) showed that the between-study variance (Level 3) was statistically significant (LRT = 53.53, *p* < 0.001), indicating significant heterogeneity across studies, whereas the within-study variance (Level 2) was not statistically significant (LRT < 0.01, *p* > 0.49), suggesting that the additional variability among multiple effect sizes within the same study was relatively limited. Although the Level 2 variance component was not significant, multiple effect sizes were extracted from individual studies, resulting in a clear hierarchical data structure. Under such circumstances, a conventional two-level model would be insufficient to account for the dependency among effect sizes. Therefore, the three-level random-effects model was considered more appropriate, as it allowed all eligible effect sizes to be retained and provided a more robust estimation of variance at different levels. Based on these findings, the three-level random-effects model was considered the primary analytical framework. The full-dataset three-level model was retained as the primary analysis of the overall intervention effect. Because the two highly influential effect sizes substantially affected the between-study heterogeneity and stability of the pooled estimate, subsequent exploratory moderator and dose–response analyses were primarily interpreted using the influence-adjusted dataset.

### Moderator analysis

In the influence-adjusted dataset, moderator analysis showed that population characteristics significantly moderated the intervention effect [*F*(2,113) = 4.69, *p* = 0.011]. Pairwise comparisons further indicated significant differences between college students with negative emotional symptoms and both other special college student populations (*t* = 2.71, *p* = 0.008) and general college students (*t* = 2.53, *p* = 0.013). By contrast, symptom category [*F*(2,113) = 1.09, *p* = 0.338], exercise modality [*F*(4,111) = 1.47, *p* = 0.217], exercise frequency [*F*(2,113) = 0.40, *p* = 0.674], session duration [*F*(3,112) = 0.69, *p* = 0.560], and intervention duration [*F*(1,114) = 1.20, *p* = 0.276] did not show significant moderating effects. Additional exploratory analyses showed significant moderating effects for geographic region [*F*(3,112) = 2.75, *p* = 0.046] and outcome measurement instrument [*F*(5,110) = 2.33, *p* = 0.047]. With Asia as the reference category, the estimated effect was significantly smaller in Europe (*b* = 0.52, *p* = 0.015), whereas North America and Other regions did not differ significantly from Asia. With the BDI family as the reference category, the estimated effects were significantly smaller for the DASS family (*b* = 0.62, *p* = 0.023) and other instruments (*b* = 0.55, *p* = 0.010), while differences for the PSS, STAI, and Zung scale families were not statistically significant. Publication year was not significantly associated with effect size in either the linear model [*β* = −0.014, 95% CI −0.039 to 0.010, *F*(1,114) = 1.32, *p* = 0.252] or the nonlinear model [*F*(3,112) = 0.63, *p* = 0.600]. Corresponding full-dataset analyses did not identify significant moderating effects for geographic region (*p* = 0.852), outcome measurement instrument (*p* = 0.558), or publication year (linear *p* = 0.052; nonlinear *p* = 0.093), indicating that the significant region- and instrument-related findings were sensitive to the two highly influential effect sizes. Detailed prespecified moderator results are presented in Table 2, and full-dataset exploratory analyses are reported in Supplementary material.

**Table 2:** Moderator analyses based on the influence-adjusted dataset.

Moderator	K, N	Hedges’ g(95% CI)	*P*	Test of moderators	Level 2	Level 3
Symptom category				*F*(2, 113) = 1.09, *p* = 0.338	2.13%	42.10%
Depression	40, 28	−0.68(−0.91, −0.44)	<0.001			
Anxiety	38, 26	−0.49(−0.72, −0.25)	<0.001			
Stress	38, 20	−0.50(−0.76, −0.25)	<0.001			
Population characteristics				F(2, 113) = 4.69, *p* = 0.011	2.02%	36.12%
General college students	68, 27	−0.47(−0.67, −0.27)	<0.001			
College students with negative emotional symptoms	34, 11	−0.96(−1.29, −0.64)	<0.001			
Other special college student populations	14, 4	−0.15(−0.65, 0.35)	0.562			
Exercise modality				*F*(4, 111) = 1.47, *p* = 0.217	2.04%	40.86%
Aerobic exercise	30, 11	−0.51(−0.84, −0.19)	0.002			
Resistance training	11, 5	−0.45(−0.96,0.07)	0.086			
Mind–body exercise	52, 18	−0.57(−0.82, −0.32)	<0.001			
Multicomponent exercise	13, 7	−0.35(−0.77,0.07)	0.106			
Other exercise modalities	10, 6	−1.05(−1.52, −0.57)	<0.001			
Exercise frequency				*F*(2, 113) = 0.40,*p* = 0.674	1.82%	42.68%
1–2 days a week	44, 13	−0.61(−0.91, −0.30)	<0.001			
3 days a week	66, 25	−0.57(−0.80, −0.34)	<0.001			
4–5 days a week	6, 4	−0.31(−0.91,0.30)	0.318			
Session duration				*F*(3, 112) = 0.69,*p* = 0.56	2.08%	42.14%
10–30 min	16, 6	−0.43(−0.87, −0.01)	0.055			
31–60 min	79, 30	−0.52(−0.73, −0.31)	<0.001			
>60 min	19, 6	−0.85(−1.30, −0.40)	<0.001			
Unclear	2, 1	−0.62(−1.75,0.51)	0.276			
Intervention duration				*F*(1, 114) = 1.20,*p* = 0.276	2.49%	42.02%
1–7 weeks	58, 18	−0.46(−0.71, −0.21)	<0.001			
8–16 weeks	58, 28	−0.63(−0.84, −0.42)	<0.001			
Geographic region				*F*(3, 112) = 2.75, *p* = 0.046	0.00%	36.69%
Asia	64, 28	−0.71(−0.92, −0.51)	<0.001			
Europe	26, 7	−0.20(−0.56,0.16)	0.275			
North America	21, 5	−0.58(−1.04, −0.11)	0.016			
Other	5, 2	0.00(−0.77,0.78)	0.996			
Outcome measurement instrument				*F*(5, 110) = 2.33, *p* = 0.047	0.00%	36.50%
BDI family	15, 11	−0.89(−1.25, −0.53)	<0.001			
DASS family	21, 6	−0.27(−0.67,0.13)	0.186			
Other instruments	31, 13	−0.34(−0.62, −0.05)	0.020			
PSS family	20, 12	−0.55(−0.87, −0.24)	0.001			
STAI family	12, 7	−0.66(−1.07, −0.25)	0.002			
Zung self-rating scales	17, 6	−0.90(−1.32, −0.48)	<0.001			
Publication year (linear)	116, 42	*β* = −0.014 (−0.039, 0.010)	0.252	*F*(1, 114) = 1.32, *p* = 0.252	0.00%	41.29%
Publication year (nonlinear)	116, 42		0.600	*F*(3, 112) = 0.63, *p* = 0.600		

K indicates the number of effect sizes and N indicates the number of studies within each subgroup. For publication year, β represents the meta-regression coefficient rather than a subgroup Hedges’ g. Geographic region and outcome measurement instrument were examined as exploratory moderators.

Within subgroup analyses, resistance training (Hedges’ *g* = −0.45, 95% CI: −0.96 to 0.07, *p* = 0.087) and multicomponent exercise (Hedges’ *g* = −0.35, 95% CI: −0.77 to 0.07, *p* = 0.106) did not reach statistical significance. Regarding exercise frequency, the subgroup of “4–5 days a week” also showed a non-significant effect (Hedges’ *g* = −0.31, 95% CI:−0.91 to 0.30, *p* = 0.318). For session duration, neither the “10–30 min” subgroup (Hedges’ *g* = −0.43, 95% CI: −0.87 to 0.01, *p* = 0.055) nor the “Unclear” subgroup (Hedges’ *g* = −0.62, 95% CI: −1.75 to 0.51, *p* = 0.276) reached statistical significance. These subgroup findings should be interpreted cautiously because several categories were represented by only a small number of studies, which may have limited statistical power and the precision of the subgroup estimates.

Using the influence-adjusted dataset, both linear and nonlinear relationships between weekly exercise duration or total exercise duration and intervention effects were examined. Because one study did not report session duration (57), it was excluded from the dose–response analysis.

For weekly exercise duration, linear and nonlinear three-level meta-regression models were fitted. In the linear model, the regression coefficient was not statistically significant [*β* = 0.0001, SE = 0.0012, *F*(1,112) = 0.003, *p* = 0.956]. In the nonlinear model, the overall test of the natural spline terms was likewise not significant [*F*(3,110) = 0.869, *p* = 0.460], indicating that no significant linear or nonlinear dose–response relationship was observed between weekly exercise duration and effect size (see Figure 4).

**Figure 4:**
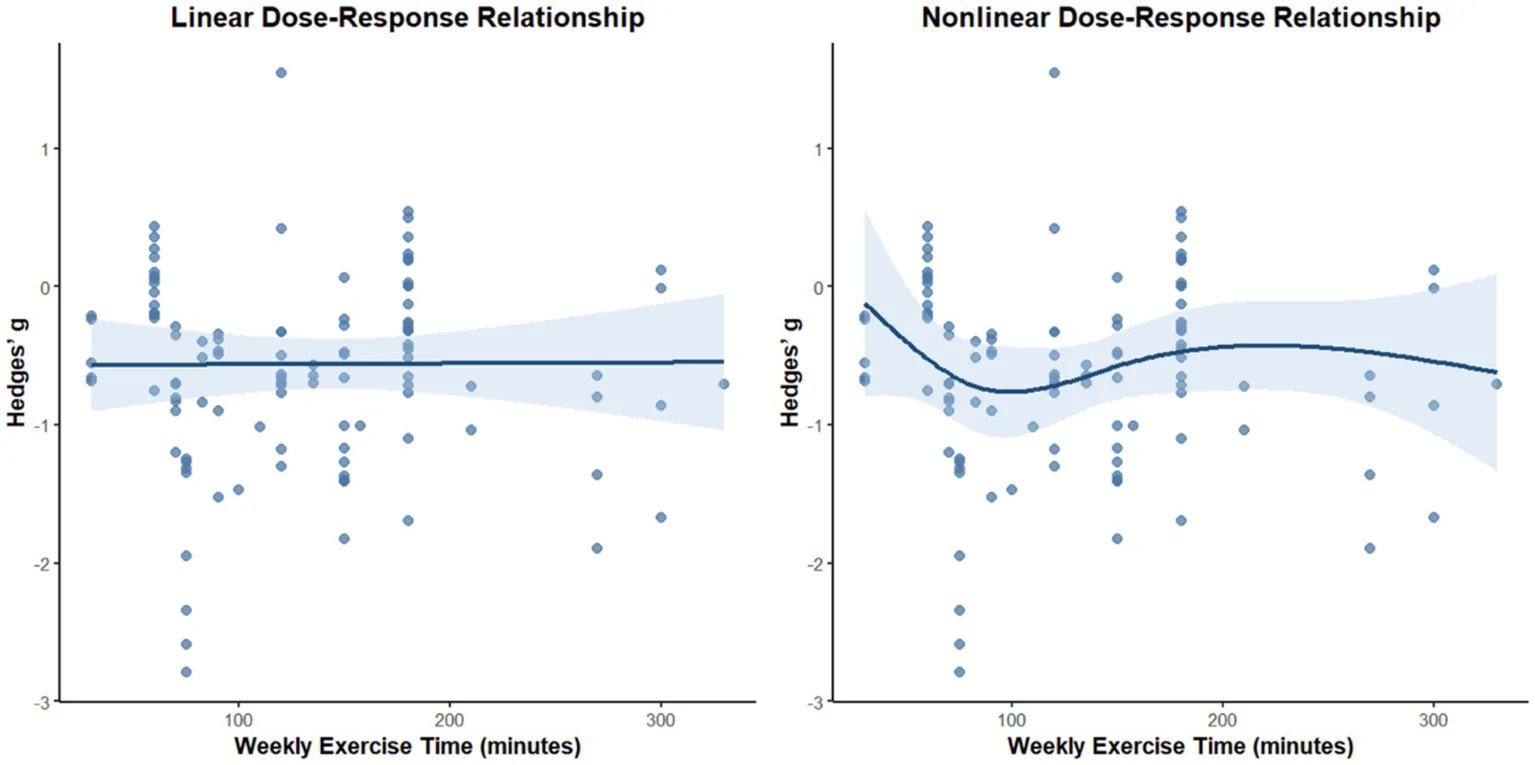
Dose–response relationship between weekly exercise duration and intervention effects.

For total exercise duration, linear and nonlinear three-level meta-regression models were also constructed. The linear model showed no statistically significant regression coefficient [*β* = 0.0000, SE = 0.0001, *F*(1,112) = 0.005, *p* = 0.945]. Similarly, in the nonlinear model, the overall test of the natural spline terms was not significant [*F*(3,110) = 0.249, *p* = 0.862], suggesting that no significant linear or nonlinear dose–response relationship was identified between total exercise duration and effect size (see Figure 5).

**Figure 5:**
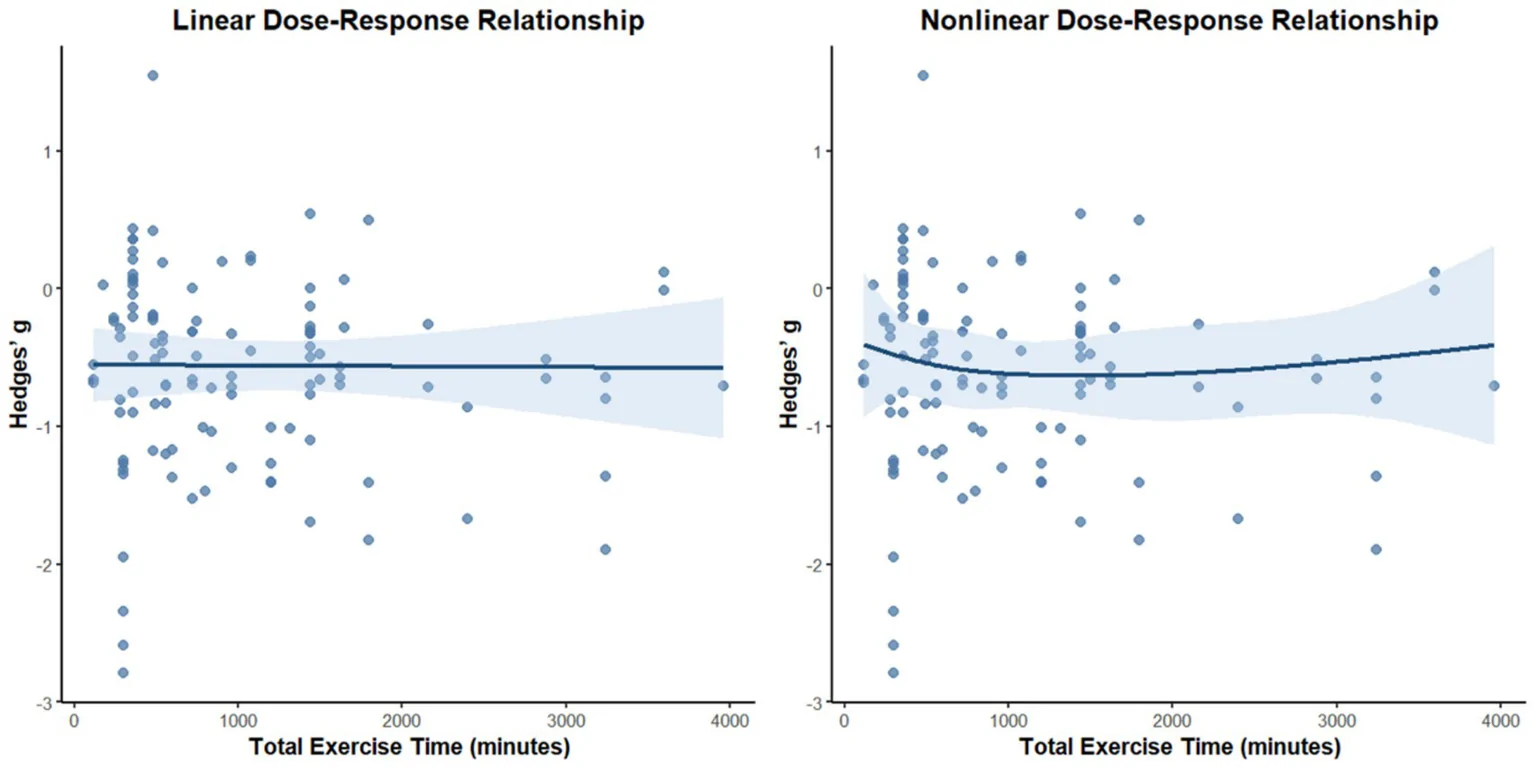
Dose–response relationship between total exercise duration and intervention effects.

### Publication bias

To assess potential publication bias, a funnel plot was first generated based on the two-level random-effects model, followed by Egger’s regression test and the Duval and Tweedie trim-and-fill procedure. The results showed that the two-level Egger’s test reached statistical significance [*t*(114) = −2.02, *p* = 0.045], suggesting the possible presence of small-study effects or funnel plot asymmetry. When the trim-and-fill method (R0) was applied, no missing effect sizes were identified for imputation, and the adjusted pooled effect (Hedges’ *g* = −0.59, 95% CI: −0.71 to −0.47) was identical to the observed estimate (Hedges’ *g* = −0.59, 95% CI: −0.71, −0.47). This suggests that the trim-and-fill procedure did not indicate a material change in the pooled effect estimate.

Because multiple effect sizes were extracted from the same studies, a multilevel Egger’s test based on CR2 robust variance estimation was additionally conducted within the three-level model. The regression coefficient for the standard error was statistically significant [slope = −2.478, SE = 1.019, *t*(18.0) = −2.43, *p* = 0.026], suggesting possible funnel plot asymmetry or small-study effects after accounting for effect-size dependency. However, the trim-and-fill procedure did not identify any missing effect sizes and did not alter the pooled estimate. These apparently different findings should not be regarded as contradictory because Egger’s regression and trim-and-fill assess different aspects of funnel plot asymmetry and may have different statistical properties, particularly in the presence of substantial heterogeneity. Finally, suspected publication bias was considered a serious concern because both the conventional and multilevel Egger’s tests suggested possible funnel plot asymmetry or small-study effects, although the trim-and-fill procedure did not materially alter the pooled estimate. Taken together, these findings suggest possible small-study effects or funnel plot asymmetry but do not conclusively demonstrate publication bias (see Figure 6).

**Figure 6:**
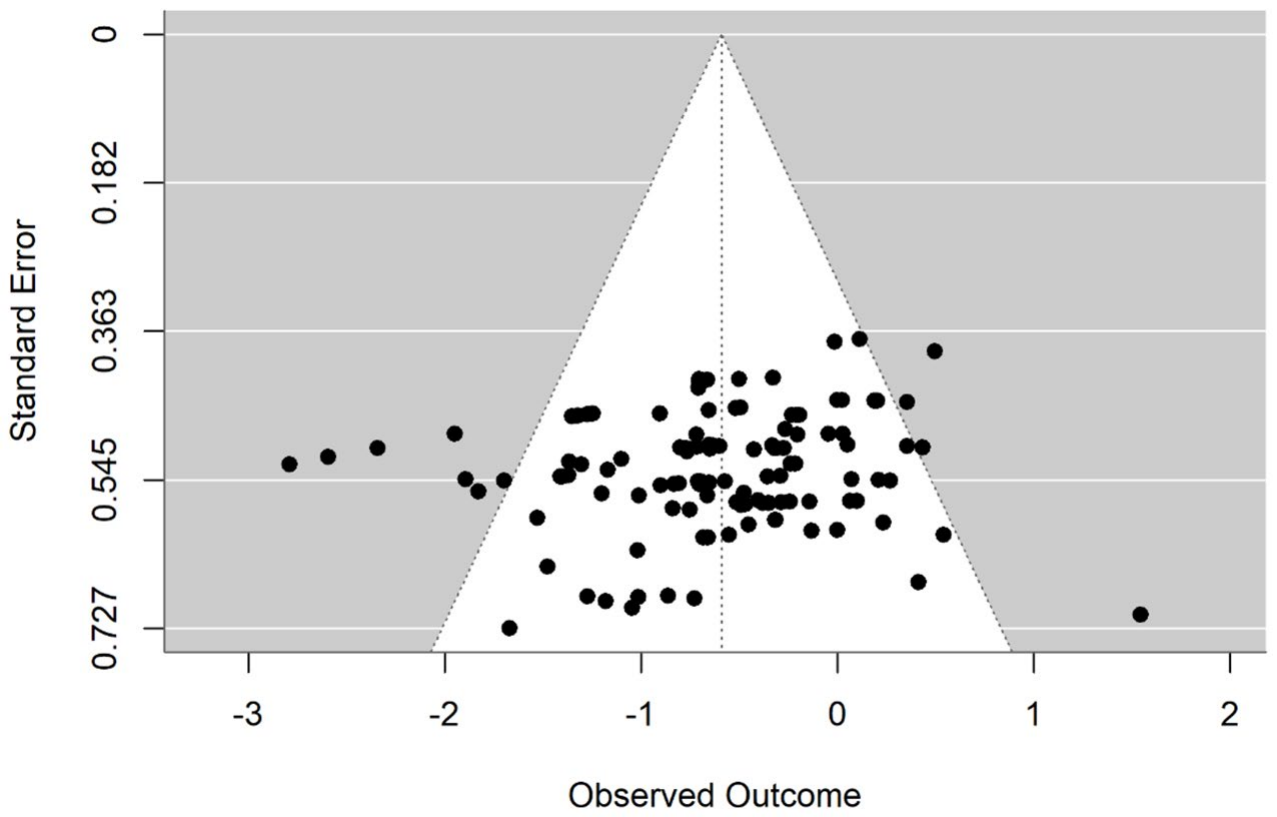
Funnel plot.

### Certainty of evidence assessed with GRADEpro

The certainty of evidence for the overall effect of physical exercise on negative emotional symptoms in college students was evaluated using GRADEpro. Because all included studies were randomized controlled trials, the evidence initially started at high certainty. The certainty was downgraded by two levels for risk of bias because most included studies were judged to be at high risk of bias, particularly for deviations from intended interventions and measurement of outcomes. It was downgraded by one level for inconsistency because substantial heterogeneity remained across studies and the between-study variance was statistically significant. No serious concerns were identified for indirectness because the populations, interventions, comparators, and outcomes were broadly consistent with the review question. The evidence was also downgraded for imprecision because the confidence interval of the full-dataset estimate was wide and crossed the null value, while the prediction interval encompassed both benefit and no effect. Finally, suspected publication bias was considered a serious concern because both the conventional and multilevel Egger’s tests suggested possible funnel plot asymmetry or small-study effects, although the trim-and-fill procedure did not materially alter the pooled estimate. Overall, the certainty of evidence was rated as very low, indicating very limited confidence in the estimated intervention effect.

## Discussion

This three-level meta-analysis systematically examined the effects of physical exercise on negative emotional symptoms in college students and explored potential moderators and dose–response relationships. In the full dataset, the overall effect did not reach statistical significance and was substantially influenced by two effect sizes from one study. After excluding these observations, the influence-adjusted analysis suggested a potential beneficial effect. However, because this finding was sensitive to the influential observations and the certainty of evidence was very low, it should be interpreted cautiously. Population characteristics, geographic region, and outcome measurement instrument emerged as potential moderators only in the influence-adjusted analysis and should therefore be regarded as exploratory. No consistent moderating or dose–response effects were identified for exercise prescription characteristics.

The potential beneficial effect observed in the influence-adjusted analysis is broadly consistent with previous research. Li et al. reported that light-, moderate-, and vigorous-intensity exercise could improve depressive, anxiety, and stress symptoms in college students, although differences between intensities were not statistically significant (13). García-Pérez et al. likewise reported beneficial effects of exercise and other non-pharmacological interventions on depression, anxiety, and stress (71). Several mechanisms may contribute to these effects. Exercise may influence neurotransmitter activity and neuroplasticity, including pathways involving serotonin, norepinephrine, and dopamine (8). It may also regulate the hypothalamic–pituitary–adrenal axis and improve sleep, thereby reducing excessive stress responses and negative mood (72). Psychosocial mechanisms may also be relevant: exercise can enhance self-efficacy, positive affect, emotion regulation, goal attainment, and peer support, which may strengthen coping resources and buffer anxiety and depression (73). These mechanisms are plausible, although they were not directly tested in the included trials (74).

In the influence-adjusted analysis, baseline population characteristics emerged as a potential moderator, with larger effects among college students with negative emotional symptoms than among general or other special populations. This is broadly consistent with evidence that responses to physical activity may vary according to psychological status and sample background (74, 75). Previous studies have also suggested that exercise may produce more pronounced effects in adolescents or college students with depressive symptoms (76). However, this moderator was not significant in the full-dataset analysis; therefore, the finding should be regarded as exploratory rather than definitive. Geographic region and outcome measurement instrument showed a similar pattern. Effects differed by region and instrument family in the influence-adjusted dataset, but these moderators were not significant in the full dataset. Such differences may reflect variation in sample composition, intervention implementation, cultural context, or psychometric properties rather than causal effects of region or measurement tool. Publication year showed no significant linear or nonlinear association with effect size.

Symptom category, exercise modality, exercise frequency, session duration, and intervention duration did not significantly moderate the intervention effect. These null findings may partly reflect limited statistical power in some subgroups and substantial heterogeneity within the same exercise categories. In addition, frequency, session duration, and intervention duration capture only selected components of the exercise prescription, while potentially important factors such as intensity, progression, and adherence were not consistently reported. Accordingly, non-significant moderator effects should not be interpreted as evidence that these factors are unimportant or that different prescriptions are equivalent. This interpretation is broadly consistent with previous reviews that did not identify a single clearly superior exercise program across mental health outcomes (10, 77). Several subgroups, particularly resistance training and the 4–5-days-per-week category, contained few studies and had relatively wide confidence intervals. Their estimates may therefore be unstable and underpowered and should be interpreted cautiously.

Neither the linear nor nonlinear three-level meta-regression identified a significant dose–response relationship for weekly exercise duration or total exercise duration. The absence of a clear pattern may be related to imprecise reporting of exercise duration and to the inability of accumulated exercise time alone to capture other relevant dose characteristics, such as intensity, progression, and adherence. This differs from some previous work suggesting that exercise intensity or modality may influence mental health outcomes (13, 78). Several factors may explain the difference. The present study integrated depression, anxiety, and stress under the broader framework of negative emotional symptoms, and these outcomes may differ in their sensitivity to exercise dose. In addition, weekly and total exercise duration were derived from session duration, frequency, and intervention duration, so incomplete reporting in the original studies may have reduced the precision of dose–response analyses. Variation in participant characteristics, intervention content, and outcome measures may also have obscured underlying dose patterns. Thus, the current evidence is insufficient to identify a stable dose–response relationship.

### Strengths, limitations, and future directions

The present study has several strengths. First, it integrated depression, anxiety, and stress within a common analytical framework while retaining symptom-specific subgroup analyses. Second, the three-level model accounted for dependence among multiple effect sizes and allowed more information from the original studies to be retained. Third, the study examined population characteristics, geographic region, measurement instrument, publication year, and dose–response relationships. Finally, robustness was evaluated using CR2 adjustment, LOSO and LOO sensitivity analyses, influence diagnostics, and complementary publication-bias analyses.

Several limitations should be considered. First, most included studies were judged to be at high risk of bias, particularly for deviations from intended interventions and outcome measurement. Second, substantial variation in participant characteristics, exercise programs, and outcome measures increased the complexity of interpretation. Exercise time, frequency, and intensity were also incompletely reported in some studies, which may have reduced the accuracy of dose–response analyses. Third, the overall findings were sensitive to two highly influential effect sizes from one study. Although their exclusion was based on objective influence diagnostics, the discrepancy between the full-dataset and influence-adjusted estimates indicates substantial uncertainty. The apparent moderation by population characteristics, geographic region, and measurement instrument was also not reproduced in the full dataset. Fourth, the review was restricted to English-language publications, so potentially eligible studies in other languages may have been missed and language bias cannot be excluded. Finally, when change-score standard deviations were unavailable, they were estimated using an assumed pre-post correlation. However, sensitivity analyses using *r* = 0.30 and *r* = 0.70 yielded the same statistical conclusion as the primary *r* = 0.50 analysis. Together with the very low certainty of evidence, these limitations warrant cautious interpretation.

Future research should use rigorous randomized designs and provide more complete reporting of randomization, attrition, and exercise prescription. Adequately powered subgroup analyses, more consistent outcome measurement, objective indicators, and longer follow-up are also needed to clarify heterogeneity, dose–response relationships, and underlying mechanisms.

## Conclusion

The full-dataset analysis did not show a statistically significant overall effect of physical exercise on negative emotional symptoms in college students. Although the influence-adjusted analysis suggested a potential benefit, the discrepancy between analyses and the very low certainty of evidence indicate substantial uncertainty. Potential moderation by population characteristics, geographic region, and outcome measurement instrument was observed only in the influence-adjusted analyses and remains exploratory. No consistent moderating or dose–response effects were identified for exercise prescription characteristics. Given the very low certainty of evidence and a prediction interval crossing the null, these findings should be regarded as suggestive rather than confirmatory.
