# Supplementary material for: Intervention effects of physical exercise on negative emotional symptoms in college students: a three-level meta-analysis
Source: Front Public Health. 2026 Oct 22;14:1911631. doi: 10.3389/fpubh.2026.1911631 (PMC13735109; doi:10.3389/fpubh.2026.1911631)
Supplement: Supplementary file 1 [file Data_Sheet_1.DOCX]

Table 1. Literature Search Strategy

| Database | Search Strategy | Number of Retrieved Studies |
| --- | --- | --- |
| the Cochrane Library |  |  |
|  | #1 MeSH descriptor: [Exercise] explode all trees | 40928 |
|  | #2 (“Physical activity” or exercise or “physical fitness” or “Yoga” or “Taichi” or “resistance training” or "physical fitness" or "strength training" or “aerobic exercise” or sport or “Pilates” or “high intensity interval training” or “HIIT”):ti,ab,kw | 202920 |
|  | #3 (“University students” or “College students” or "higher education" or “university” or undergraduate ):ti,ab,kw | 95608 |
|  | #4 MeSH descriptor: [Mental Health] explode all trees | 3579 |
|  | #5 (“mental health” or “psychological health” or “mental” or “anxiety” or depression or wellbeing or stress or coping or “social isolation” or distress or loneliness or sleep):ti,ab,kw | 324447 |
|  | #6 MeSH descriptor: [Randomized Controlled Trial] explode all trees | 34 |
|  | #7 (“randomized controlled trial” or parallel or single blind or blind or randomized or controlled or trial or “RCT”):ti,ab,kw | 1850803 |
|  | #8 #1 OR #2 | 300165 |
|  | #9 #4 OR #5 | 324447 |
|  | #10 #6 OR #7 | 1850903 |
|  | #3 AND #8 AND #9 AND #10 | Trials: 3241 |
| Embase |  |  |
|  | ('university students':ti,ab OR 'college students'/exp OR 'college students':ti,ab OR 'higher education':ti,ab OR 'university':ti,ab OR 'undergraduate':ti,ab) AND ('exercise'/exp OR 'physical activity':ti,ab OR 'exercise':ti,ab OR 'yoga':ti,ab OR 'taichi':ti,ab OR 'resistance training':ti,ab OR 'physical fitness':ti,ab OR 'strength training':ti,ab OR 'aerobic exercise':ti,ab OR 'sport':ti,ab OR 'pilates':ti,ab OR 'high intensity interval training':ti,ab OR 'hiit':ti,ab) AND ('mental health'/exp OR 'mental health':ti,ab OR 'psychological health':ti,ab OR 'mental':ti,ab OR 'anxiety':ti,ab OR 'depression':ti,ab OR 'wellbeing':ti,ab OR 'stress':ti,ab OR 'coping':ti,ab OR 'social isolation':ti,ab OR 'distress':ti,ab OR 'loneliness':ti,ab OR 'sleep':ti,ab) AND ('randomized controlled trial'/exp OR 'parallel':ti,ab OR 'single blind':ti,ab OR 'blind':ti,ab OR 'randomized':ti,ab OR 'controlled':ti,ab OR 'trial':ti,ab OR 'rct':ti,ab) | 3553 |
| Web of Science |  |  |
|  | #1 TS=(“University students” or “College students” or "higher education" or “university” or undergraduate ) | 609205 |
|  | #2 TS=(“Physical activity” or exercise or “physical fitness” or “Yoga” or “Taichi” or “resistance training” or "physical fitness" or "strength training" or “aerobic exercise” or sport or “Pilates” or “high intensity interval training” or “HIIT”) | 625002 |
|  | #3 TS=(“mental health” or “psychological health” or “mental” or “anxiety” or depression or wellbeing or stress or coping or “social isolation” or distress or loneliness or sleep) | 3046905 |
|  | #4 TS=(“randomized controlled trial” or parallel or single blind or blind or randomized or controlled or trial or “RCT”) | 6267813 |
|  | #5 #1 AND #2 AND #3 AND #4 | 3340 |
| Scopus |  |  |
|  | #1 "University students" OR "College students" OR "higher education" OR "university" OR undergraduate |  |
|  | #2 "Physical activity" OR exercise OR "physical fitness" OR "Yoga" OR "Taichi" OR "resistance training" OR "physical fitness" OR "strength training" OR "aerobic exercise" OR sport OR "Pilates" OR "high intensity interval training" OR "HIIT" |  |
|  | #3 "mental health" OR "psychological health" OR "mental" OR "anxiety" OR depression OR wellbeing OR stress OR coping OR "social isolation" OR distress OR loneliness OR sleep |  |
|  | #4 "randomized controlled trial" OR parallel OR single blind OR blind OR randomized OR controlled OR trial OR "RCT" |  |
|  | #5 #1 AND #2 AND #3AND #4 | A total of 2,173 records were identified through searches conducted in the title, abstract, and keyword fields. |
| Pubmed |  |  |
|  | #1 "Exercise"[Mesh] | 277700 |
|  | #2 "Physical activity"[Title/Abstract] OR exercise[Title/Abstract] OR "physical fitness"[Title/Abstract] OR "Yoga"[Title/Abstract] OR "Taichi"[Title/Abstract] OR "resistance training"[Title/Abstract] OR "physical fitness"[Title/Abstract] OR "strength training"[Title/Abstract] OR "aerobic exercise"[Title/Abstract] OR sport[Title/Abstract] OR "Pilates"[Title/Abstract] OR "high intensity interval training"[Title/Abstract] OR "HIIT"[Title/Abstract] | 563704 |
|  | #3 "University students"[Title/Abstract] OR "College students"[Title/Abstract] OR "higher education"[Title/Abstract] OR "university"[Title/Abstract] OR undergraduate[Title/Abstract] | 602015 |
|  | #4 "Mental Health"[Mesh] | 75021 |
|  | #5 "mental health"[Title/Abstract] OR "psychological health"[Title/Abstract] OR "mental"[Title/Abstract] OR "anxiety"[Title/Abstract] OR depression[Title/Abstract] OR wellbeing[Title/Abstract] OR stress[Title/Abstract] OR coping[Title/Abstract] OR "social isolation"[Title/Abstract] OR distress[Title/Abstract] OR loneliness[Title/Abstract] OR sleep[Title/Abstract] | 2592126 |
|  | #6 randomized controlled trial[Publication Type] | 649393 |
|  | #7 "randomized controlled trial"[Title/Abstract] OR parallel[Title/Abstract] OR single blind[Title/Abstract] OR blind[Title/Abstract] OR randomized[Title/Abstract] OR controlled[Title/Abstract] OR trial[Title/Abstract] OR "RCT"[Title/Abstract] | 2278736 |
|  | #8 #1 OR #2 | 656328 |
|  | #9 #4 OR #5 | 2599309 |
|  | #10 #6 OR #7 | 2443867 |
|  | #11 #8 AND #3 AND #9 AND #10 | 1263 |

**Statistical analysis**

Because some of the included primary studies reported multiple negative emotional symptoms, dependency among effect sizes within the same study was likely to occur, making the independence assumption required by conventional two-level meta-analysis difficult to satisfy. Therefore, this study used a three-level random-effects meta-analytic model to simultaneously incorporate multiple effect sizes from the same study. In a three-level meta-analysis, the total variance is partitioned into sampling variance (Level 1), within-study variance (Level 2), and between-study variance (Level 3). This approach allows the inclusion of multiple relevant effect sizes from the original studies and thereby improves statistical efficiency (Assink and Wibbelink, 2016). All three-level meta-analyses were conducted in R using the metafor package, following the procedures described by Assink (Assink and Wibbelink, 2016) and Liu (Liu et al., 2026).

First, a three-level random-effects model without moderators was fitted to estimate the overall pooled effect. Effect sizes were expressed as Hedges’ g, together with their 95% confidence intervals and 95% prediction intervals. Negative effect sizes indicated a reduction in the level of negative emotions among college students following exercise intervention, reflecting an improvement in outcomes. Overall heterogeneity was then assessed using Cochran’s Q test. In addition, the variance components of the three-level model were decomposed by level, and likelihood ratio tests (LRTs) were used to determine whether the Level 2 and Level 3 variance components were statistically significant (Cheung, 2014). For comparison with traditional approaches, results from a conventional two-level random-effects model were also reported as supplementary reference; however, the primary interpretation was based on the three-level model.

Based on the heterogeneity assessment, moderator analyses were further conducted to explore potential sources of heterogeneity. Categorical moderators were examined by entering the relevant variables into three-level meta-regression models; when the overall moderator test was statistically significant, pairwise comparisons between subgroups were performed. Continuous moderators were analyzed using three-level meta-regression. To further examine exercise dose characteristics, two continuous variables were constructed for dose–response analysis: weekly exercise duration and total exercise duration. Weekly exercise duration was calculated as session duration × exercise frequency per week, and total exercise duration was calculated as weekly exercise duration × intervention duration. For studies reporting only a range of session duration rather than an exact value, the midpoint of the interval was used as an approximate estimate (Liu et al., 2026). To investigate dose–response relationships, both linear and nonlinear three-level meta-regression models were fitted. In the linear analyses, exercise time variables were directly entered into the model as continuous predictors. In the nonlinear analyses, natural spline functions (natural spline, df = 3) were applied to model exercise time and test for potential nonlinear dose–response relationships (Yu et al., 2024).

To examine the robustness of the findings, sensitivity analyses and influence analyses were performed. Sensitivity analyses included CR2-based robust variance estimation as well as leave-one-study-out and leave-one-effect-out analyses (Viechtbauer and Cheung, 2010). CR2 robust variance estimation was used to adjust standard errors and confidence intervals while accounting for the dependence among effect sizes within studies, with degrees of freedom estimated using the Satterthwaite approximation (Hedges et al., 2010). In the leave-one-study-out and leave-one-effect-out analyses, the main model was refitted after omitting one study or one effect size at a time, respectively, to assess the influence of individual studies or effect sizes on the overall results. In addition, influence analyses were conducted to identify statistically extreme and highly influential effect sizes. Effect sizes were not excluded solely because of their magnitude or because of their impact on statistical significance. When an effect size was simultaneously identified as statistically extreme and exerted a disproportionate influence on the pooled estimate, confidence interval, prediction interval, and heterogeneity structure, an additional influence-adjusted analysis was performed after excluding these observations. The model based on the full dataset was retained as the primary analysis of the overall pooled effect. Given the substantial influence of these observations on model stability and heterogeneity, subsequent subgroup, moderator, and dose-response analyses were primarily interpreted using the influence-adjusted dataset. Corresponding analyses based on the full dataset were additionally conducted to assess the sensitivity of these exploratory findings.

To assess potential publication bias, funnel plots were first generated under the two-level random-effects model, followed by Egger’s regression test and the Duval and Tweedie trim-and-fill procedure (Egger et al., 1997). Given the dependence structure arising from multiple effect sizes within the same study, a multilevel Egger test was further conducted by including the standard error as a moderator in the three-level model, with CR2 robust variance estimation and Satterthwaite-adjusted degrees of freedom applied to determine whether small-study effects or funnel plot asymmetry remained after accounting for effect size dependency. If the pooled effect size changed only minimally before and after trim-and-fill adjustment, the impact of potential publication bias on the overall conclusions was considered limited (Duval and Tweedie, 2000).

The certainty of evidence was evaluated using the GRADE approach and implemented in GRADEpro software (Atkins et al., 2004; Warneke et al., 2024; Hu et al., 2025). Because all included studies were randomized controlled trials, the certainty of evidence was initially rated as high. It was then assessed across five domains: risk of bias, inconsistency, indirectness, imprecision, and publication bias, and finally rated as high, moderate, low, or very low (Liu et al., 2024). Specifically, risk of bias was judged according to the RoB 2.0 assessment results; inconsistency was evaluated primarily on the basis of heterogeneity tests, variance decomposition, and sensitivity analyses; imprecision was judged according to whether the 95% CI of the pooled effect crossed the null value and by the width of the interval; and publication bias was assessed comprehensively using funnel plots, Egger’s test, and trim-and-fill results.

Table 2. Intervention Characteristics and Outcome Measures of the Included Studies

| Included studies | Intervention characteristics (exercise modality/session duration/frequency/intervention duration/intensity/control condition) | Outcome measures |
| --- | --- | --- |
| Abavisani et al., 2019 | Pilates/60 minutes/2 times a week/8 weeks/Unclear/Regular activities | SAQ |
| Akandere et al., 2011 | Dance/110 minutes/3 times a week/12 weeks/Unclear/Regular activities | BDI |
| Alsaraireh et al., 2017 | Aerobics and strength exercises/60 minutes/3 times a week/10 weeks/Unclear/Mindfulness meditation | CES-D |
| Asci et al., 2003 | One aerobic two step dance per week /50 minutes/3 times a week/10 weeks/60-80%HR reserve/Normal daily activities | STAI |
| Barğı et al., 2022 | Physical exercises/30-60 minutes/3-5 times a week/4 weeks/Unclear/Regular activities | BDI, BAI, |
| De Vries et al., 2017 | Running/60 minutes/3 times a week/6 weeks/ Low intensity/Wait list | Stress |
| Dinani et al., 2019 | Taichi/40 minutes/3 times a week/8 weeks/Unclear/Regular activities | DASS-42 |
| Eather et al., 2019 | HIIT/8-12 minutes/3 times a week/8 weeks/85% Max HR/Wait list | PSS, STAI, |
| Ghorbani et al., 2014 | Running and rope skipping/60 minutes/3 times a week/6 weeks/unclear/Usual activities | GHQ-28 mental health questionnaire |
| Hemat-Far et al.,2012 | Aerobic Exercises/40-60 minutes/3 times a week/8 weeks/60-65%HRmax/Regular activities | BDI |
| Kim et al., 2004 | Meridian Exercise/30 minutes/2 times a week/6weeks/Unclear/Regular activities | SAI, DSI |
| Kim et al., 2013 | Kouk Sun Do/70 minutes/3 times a week/4 weeks/Unclear/Wait list | BDI-II, STAI |
| Li et al., 2015 | Baduanjin training/60 minutes/5 times a week/12 weeks/Unclear/Regular activities | CPSS |
| Li et al., 2022a | Resistance training/50 minutes/2 times a week/8 weeks/70%RM/Regular activities | SAS |
| López-Rodríguez et al., 2017 | Biodanza/90 minutes/ 1 time a week/4 week/Wait list | CES-D, PSS |
| Paolucci et al., 2018 | High-intensity interval training/ 20 minutes/3 times a week/6 weeks/60-s high intensity intervals at 80% maximum wattage alternating with ten 60-s active recovery intervals at 30% of their maximum wattage/ Remain sedentary;  Moderate continuous training/ 27.5 minutes/3 times a week/6 weeks/40% of maximum wattage/ Remain sedentary | BDI, BAI, PSS |
| Papp et al., 2019 | Modern hatha yoga/60 minutes/1 time a week/6 weeks/Intensity/Regular activities | PSS, HADS |
| Philippot et al., 2022 | High-Intensity Interval Training/10 minutes/3 times a week/4 weeks/Alternating between High  intensity exercises (30s) ≥6 RPE and active recovery exercises (30s) ≤4 RPE/ Usual activities | DASS-21, |
| Roth and Holmes, 1987 | Aerobic Exercise Training/30 minutes/5 times a week /11 weeks/75%HRmax/Usual activities； | BDI, STAI |
| Sadeghi et al., 2016 | Aerobic exercise/50-60 minutes/2 times a week/12 weeks/60-80%HRmax/ Group discussion | BDI |
| Saltan and Ankaralı, 2021 | Therapeutic exercise(Mix)/Unclear/3times a week/12 weeks/Low -to moderate‐intensity/no regular physical activity;  Pilates/Unclear/3times a week/12 weeks/Low -tomoderate‐intensity/no regular physical activity; | BDI |
| Wan Yunus et al., 2020 | Exergame/30 minutes/3 times a week/6 weeks/Unclear/Daily life | DASS-21 |
| Xiao et al., 2021 | Basketball exercise/90 minutes/3 times a week/12 weeks/Moderate intensity/Usual activities;  Baduanjin exercise/90 minutes/3 times a week/12 weeks/Moderate intensity/Usual activities; | SRAS, PSS |
| Yiğiter and Hardee, 2017 | Tennis exercise/60 minutes/3 times a week/8 weeks/Unclear/Daily activities | BDI |
| Zhang JW et al., 2023 | Taichi/60 minutes/5 times a week/8 weeks/Unclear/Daily activities | SAS, SDS |
| Zhang and Jiang, 2023 | Baduanjin/60 minutes/3 times a week/12 weeks/Unclear/Daily activities | SCL-90 |
| Zhao et al., 2023 | Aerobic exercise/40-60 minutes/3 times a week/12 weeks /50-90%HRR/Wait list;  Resistance exercise/40-60 minutes/3 times a week/12 weeks /20RM/Wait list; | SDS |
| Zheng et al., 2015 | Taichi/60 minutes/5 times a week/12 weeks/Unclear/Daily activities | PSS |
| Ji et al., 2022 | Team Sports(basketball games)/60 minutes/2 times a week/6 weeks/Unclear/Daily activities;  Individual sports (running exercises and strength training)/60 minutes/2 times a week/6 weeks/Unclear/Daily activities; | SAS |
| Gallego et al., 2015 | Physical exercise/60 minutes/1 time a week/8 weeks/Unclear/Daily activities; | DASS-21 |
| Zhang et al., 2024 | Aerobic exercise/60 minutes/3 times a week/8 weeks/Unclear/Wait list | SAS,SDS |
| Brandão et al., 2024 | Yoga/60 minutes/1 time a week/6 weeks/Unclear/Autogenic training;  Yoga/60 minutes/1 time a week/6 weeks/Unclear/Daily activities; | DASS-21 |
| Calazans de Lira et al., 2024 | Strength Training/60 minutes/2 times a week/4 weeks/Unclear/Daily activities | HAD |
| Falsafi et al., 2016 | Yoga/75 minutes/1 time a week/8 weeks/Unclear/  Daily activities | BDI, HAS, SLSI |
| Castellote-Caballero et al., 2024 | Yoga/60 minutes/2 times a week/12 weeks/Unclear/Daily activities | PSS,STAI, |
| Suwannakul et al., 2025 | Yoga//3 times a week/8 weeks/Unclear/Daily activities | PSS |
| Lin Latt et al., 2024 | Resistance Training/45-60 minutes/3 times a week/5 weeks/70-85% 1RM/Wait list | PSS |
| Zhang et al., 2018 | Taichi/90 minutes/2 times a week/8 weeks/Daily activities | PHQ-9,PSS |
| Cui et al., 2025 | Resistance training/60 minutes/3 times a week/8 weeks/60-70% 1RM/Daily activities;  Time-restricted fasting plus resistance training/60 minutes/3 times a week/8 weeks/60-70% 1RM/Time-restricted fasting; | DASS-21 |
| Wang et al., 2024b | Baduanjin/60 minutes/3 times a week/16weeks/Unclear/Daily activities | SCL-90 |
| Wang et al., 2024a | Taichi/45 minutes/3 times a week/12 weeks/Unclear/Daily activities | STAI,SAI,T-AI |
| Yun et al., 2025 | Taekwondo/30 minutes/3 times a week/8 weeks/64-76%HRmax/Reading | BDI |
| Aslan et al., 2025 | Laughter Yoga/30-45 minutes/2 times a week/4weeks/Unclear/Regular activities | STAI,  PSSNS |

SAQ for Spielberger Anxiety Questionnaire; BDI/BDI-II for Beck Depression Scale; CES-D for The Center for Epidemiologic Studies Depression Scale; STAI for State-trait anxiety inventory;PSSNS for Perceived Stress Scale for Nursing Students;BAI for Beck Anxiety Inventory;DSI for Depression Status Inventory scale; PSS for Perceived Stress Scale; HADS for the Hospital Anxiety and Depression Scale；SRAS for Self-Rating Anxiety Scale; SAS for self-rating anxiety scale, SDS for self-rating depression scale；SCL-90 for symptom checklist-90; HRR for heart rate reserve;RM for repetitions maximum; HAD for Hospital Anxiety and Depression scale; SLSI for Student-Life Stress Inventory; PHQ-9 for Nine-item patient health questionnaire depression scale

Figure 1. Risk of Bias Assessment Based on RoB 2.0

**Heterogeneity Analysis Based on the Full Dataset**

In the full dataset, 43 studies comprising 118 effect sizes were included. Cochran’s Q test indicated significant overall heterogeneity (Q(117) = 978.69, P < 0.001). Variance decomposition showed that sampling variance accounted for 0.83% of the total variance, within-study variance (Level 2) for approximately 0.00%, and between-study variance (Level 3) for 99.17%. The likelihood ratio test showed that the Level 3 variance component was statistically significant (LRT = 313.48, P < 0.001), whereas the Level 2 variance component was not statistically significant (LRT = 0.00, P = 1.000).

**Supplementary Table 3. Moderator Analyses Based on the Full Dataset**

| Moderator | K, N | Hedges’ g (95% CI) | P | Test of Moderators |
| --- | --- | --- | --- | --- |
| Symptom category |  |  |  | F(2,115) = 0.82, P = 0.443 |
| Depression | 41, 29 | -1.59 (-3.33, 0.15) | 0.074 |  |
| Anxiety | 39, 27 | -1.41 (-3.15, 0.34) | 0.112 |  |
| Stress | 38, 20 | -1.41 (-3.15, 0.34) | 0.114 |  |
| Population characteristics |  |  |  | F(2,115) = 0.17, P = 0.842 |
| General college students | 70, 28 | -1.84 (-4.04, 0.36) | 0.101 |  |
| College students with negative emotional symptoms | 34, 11 | -1.02 (-4.53, 2.49) | 0.566 |  |
| Other special college student populations | 14, 4 | -0.26 (-6.07, 5.56) | 0.930 |  |
| Exercise modality |  |  |  | F(4,113) = 0.61, P = 0.656 |
| Aerobic exercise | 30, 11 | -1.29 (-3.16, 0.58) | 0.175 |  |
| Resistance training | 11, 5 | -1.07 (-3.09, 0.94) | 0.294 |  |
| Mind-body exercise | 54, 19 | -1.43 (-3.22, 0.36) | 0.116 |  |
| Multicomponent exercise | 13, 7 | -1.75 (-3.71, 0.21) | 0.080 |  |
| Other exercise modalities | 10, 6 | -2.07 (-3.99, -0.16) | 0.034 |  |
| Exercise frequency |  |  |  | F(2,115) = 1.06, P = 0.350 |
| 1–2 days a week | 46, 14 | -3.31 (-6.36, -0.27) | 0.033 |  |
| 3 days a week | 66, 25 | -0.63 (-2.91, 1.65) | 0.584 |  |
| 4–5 days a week | 6, 4 | -0.36 (-6.06, 5.33) | 0.900 |  |
| Session duration |  |  |  | F(3,114) = 0.06, P = 0.980 |
| 10–30 minutes | 18, 7 | -1.51 (-3.60, 0.58) | 0.156 |  |
| 31–60 minutes | 79, 30 | -1.63 (-3.59, 0.33) | 0.101 |  |
| >60 minutes | 19, 6 | -0.85 (-5.62, 3.93) | 0.727 |  |
| Unclear | 2, 1 | -0.62 (-12.33, 11.08) | 0.916 |  |
| Intervention duration |  |  |  | F(1,116) = 0.20, P = 0.655 |
| 1–7 weeks | 60, 19 | -1.41 (-3.18, 0.35) | 0.116 |  |
| 8–16 weeks | 58, 28 | -1.52 (-3.27, 0.23) | 0.087 |  |
| Geographic region |  |  |  | F(3,114) = 0.26, P = 0.852 |
| Asia | 66, 29 | -2.03 (-4.21, 0.15) | 0.068 |  |
| Europe | 26, 7 | -0.23 (-4.65, 4.20) | 0.919 |  |
| North America | 21, 5 | -0.65 (-5.90, 4.59) | 0.806 |  |
| Other | 5, 2 | 0.01 (-8.28, 8.30) | 0.998 |  |
| Outcome measurement instrument |  |  |  | F(5,112) = 0.79, P = 0.558 |
| BDI family | 15, 11 | -2.03 (-3.97, -0.09) | 0.040 |  |
| DASS family | 21, 6 | -0.28 (-4.97, 4.40) | 0.905 |  |
| Other instruments | 32, 14 | -1.64 (-3.56, 0.29) | 0.095 |  |
| PSS family | 20, 12 | -1.49 (-3.41, 0.43) | 0.128 |  |
| STAI family | 13, 8 | -1.87 (-3.83, 0.09) | 0.061 |  |
| Zung self-rating scales | 17, 6 | -1.33 (-3.42, 0.77) | 0.212 |  |

**Note.** K indicates the number of effect sizes and N indicates the number of studies within each subgroup. For geographic region, Asia included China, South Korea, Malaysia, Thailand, Iran, Turkey, and Jordan; Europe included the Netherlands, Spain, Sweden, Belgium, and Portugal; North America included the United States and Canada; and Other included Australia and Brazil. All moderator tests were conducted using three-level random-effects meta-regression models based on the full dataset.

Publication year was additionally examined as a continuous exploratory moderator. The linear three-level meta-regression showed a positive but non-significant association with effect size (β = 0.2351, SE = 0.1199, F(1,116) = 3.85, P = 0.052; 95% CI -0.0023 to 0.4726). The nonlinear natural-spline model was also not statistically significant (F(3,114) = 2.19, P = 0.093). Thus, geographic region, outcome measurement instrument, and publication year did not significantly explain between-study heterogeneity in the full dataset.

**Sensitivity Analysis of Dose–Response Relationships Based on the Full Dataset**

For weekly exercise duration, the linear three-level meta-regression model showed no statistically significant association with effect size (β = 0.0071, SE = 0.0105, F(1,114) = 0.455, P = 0.501). Similarly, the nonlinear natural spline model was not statistically significant (F(3,112) = 0.719, P = 0.543).

For total exercise duration, the linear three-level meta-regression model also showed no statistically significant association with effect size (F(1,114) = 0.007, P = 0.932). Likewise, the nonlinear natural spline model was not statistically significant (F(3,112) = 0.060, P = 0.981). These findings were consistent with the influence-adjusted analyses and indicated no evidence of either linear or nonlinear dose-response relationships for weekly or total exercise duration.

Assink, M., and Wibbelink, C.J. (2016). Fitting three-level meta-analytic models in R: A step-by-step tutorial. *The Quantitative Methods for Psychology* 12(3)**,** 154-174.

Atkins, D., Best, D., Briss, P.A., Eccles, M., Falck-Ytter, Y., Flottorp, S., et al. (2004). Grading quality of evidence and strength of recommendations. *Bmj* 328(7454)**,** 1490. doi: 10.1136/bmj.328.7454.1490.

Cheung, M.W. (2014). Modeling dependent effect sizes with three-level meta-analyses: a structural equation modeling approach. *Psychol Methods* 19(2)**,** 211-229. doi: 10.1037/a0032968.

Duval, S., and Tweedie, R. (2000). Trim and fill: A simple funnel-plot-based method of testing and adjusting for publication bias in meta-analysis. *Biometrics* 56(2)**,** 455-463. doi: 10.1111/j.0006-341x.2000.00455.x.

Egger, M., Davey Smith, G., Schneider, M., and Minder, C. (1997). Bias in meta-analysis detected by a simple, graphical test. *Bmj* 315(7109)**,** 629-634. doi: 10.1136/bmj.315.7109.629.

Hedges, L.V., Tipton, E., and Johnson, M.C. (2010). Robust variance estimation in meta-regression with dependent effect size estimates. *Res Synth Methods* 1(1)**,** 39-65. doi: 10.1002/jrsm.5.

Hu, Y., Gu, S., Bu, Z., Liu, Z., Dong, J., Shi, J., et al. (2025). Effect of exercise for patients with advanced lung cancer and cancer-related fatigue: A systematic review and meta-analysis. *J Sport Health Sci* 14**,** 101017. doi: 10.1016/j.jshs.2024.101017.

Liu, C., Chen, R., Jia, S., Guo, Z., Wei, C., and Wang, X. (2026). Effects of exercise on cognitive function in patients with depression: a three-level meta-analysis with dose-response and exploratory mediation analyses. *International Journal of Behavioral Nutrition and Physical Activity*. doi: 10.1186/s12966-026-01902-3.

Liu, C., Chen, R., Yun, S.M., and Wang, X. (2024). Intervention effect of exercise on working memory in patients with depression: a systematic review. *PeerJ* 12**,** e17986. doi: 10.7717/peerj.17986.

Viechtbauer, W., and Cheung, M.W. (2010). Outlier and influence diagnostics for meta-analysis. *Res Synth Methods* 1(2)**,** 112-125. doi: 10.1002/jrsm.11.

Warneke, K., Plöschberger, G., Lohmann, L.H., Lichtenstein, E., Jochum, D., Siegel, S.D., et al. (2024). Foam rolling and stretching do not provide superior acute flexibility and stiffness improvements compared to any other warm-up intervention: A systematic review with meta-analysis. *J Sport Health Sci* 13(4)**,** 509-520. doi: 10.1016/j.jshs.2024.01.006.

Yu, C., Wang, T., Gao, Y., Jiao, Y., Jiang, H., Bian, Y., et al. (2024). Association between physical activity and risk of gastroesophageal reflux disease: A systematic review and meta-analysis. *J Sport Health Sci* 13(5)**,** 687-698. doi: 10.1016/j.jshs.2024.03.007.
